# CCTop: An Intuitive, Flexible and Reliable CRISPR/Cas9 Target Prediction Tool

**DOI:** 10.1371/journal.pone.0124633

**Published:** 2015-04-24

**Authors:** Manuel Stemmer, Thomas Thumberger, Maria del Sol Keyer, Joachim Wittbrodt, Juan L. Mateo

**Affiliations:** Centre for Organismal Studies (COS), Heidelberg University, Heidelberg, Germany; NIH, UNITED STATES

## Abstract

Engineering of the CRISPR/Cas9 system has opened a plethora of new opportunities for site-directed mutagenesis and targeted genome modification. Fundamental to this is a stretch of twenty nucleotides at the 5’ end of a guide RNA that provides specificity to the bound Cas9 endonuclease. Since a sequence of twenty nucleotides can occur multiple times in a given genome and some mismatches seem to be accepted by the CRISPR/Cas9 complex, an efficient and reliable *in silico* selection and evaluation of the targeting site is key prerequisite for the experimental success. Here we present the CRISPR/Cas9 target online predictor (CCTop, http://crispr.cos.uni-heidelberg.de) to overcome limitations of already available tools. CCTop provides an intuitive user interface with reasonable default parameters that can easily be tuned by the user. From a given query sequence, CCTop identifies and ranks all candidate sgRNA target sites according to their off-target quality and displays full documentation. CCTop was experimentally validated for gene inactivation, non-homologous end-joining as well as homology directed repair. Thus, CCTop provides the bench biologist with a tool for the rapid and efficient identification of high quality target sites.

## Introduction

Targeted genome editing has become available to literally all (model) organisms with the emergence of engineered nucleases like transcription activator-like effector nucleases (TALEN) [1], Zinc-finger nucleases (ZFN) [2] or the RNA guided nucleases [3] facilitating the introduction of a double strand break (DSB) at any locus of choice [4–9].

For targeted genome editing the **c**lustered **r**egularly **i**nterspaced **s**hort **p**alindromic **r**epeats (CRISPR)/ CRISPR associated 9 (Cas9) system, initially discovered as ‘immune response’ in archaea and bacteria, has rapidly evolved as the tool of choice [10–12]. A single guide RNA (sgRNA) provides specificity and targets the Cas9 endonuclease to introduce a DSB at the site determined by the sgRNA [3]. A target sequence is characterized by a stretch of twenty nucleotides followed by a **p**rotospacer **a**djacent **m**otif (PAM; NRG in case of Cas9) [13]. With these straightforward design criteria, targeting of any locus in a given genome appears feasible. However, since a stretch of twenty nucleotides can occur multiple times in a given genome and some mismatches seem to be accepted by the CRISPR/Cas9 system [3,13–15], an efficient and reliable *in silico* selection and evaluation of the targeting site is key prerequisite for the experimental success.

To this end, already a number of online sgRNA target finding and evaluation tools like CRISPR Design [13], E-CRISP [16] or CHOPCHOP [17] have been presented. For the selection of target sites they all have their individual strengths and limitations. In particular some run on restrictive sets of parameters, take too few mismatches into account for off-target search, lack full documentation about potential off-target sites, or have a limited list of target genomes.

To provide the bench biologist with a tool for the rapid and efficient identification of high quality target sites, we have combined the strengths and overcome the limitations in the newly developed **C**RISPR/**C**as9 **T**arget **o**nline **p**redictor (CCTop). We provide a growing range of model system genomes that can be analyzed via an intuitive graphical user interface for data entry. The output presents all the relevant information at a glance. CCTop has a reasonable number of options to provide the beginner with a list of top candidates (and the corresponding oligonucleotide sequences for cloning) and the expert with flexible options and a complete documentation. Thus, the user is well informed for selecting the target site of choice. Here we present CCTop as an experimentally validated system for the rapid selection of high quality target sites for gene inactivation, non-homologous end-joining as well as homology directed repair.

## Materials and Methods

### CCTop

CCTop is a web tool composed of html pages and CGI scripts (http://crispr.cos.uni-heidelberg.de). The main processing steps are implemented in python (S1 Fig).

### Off-target search

The search of off-target sites is carried out using Bowtie [18] version 0.12.7. Advantage has been taken of the seed used by Bowtie to search for matches and was linked to our definition of the sgRNA core plus the PAM. However prior to Bowtie based alignment, the sgRNA target sequence has to be reverse complemented as a prerequisite of Bowtie’s alignment procedure, which only starts at the 5’ end. With this modification Bowtie is invoked with the following parameters:-a, -n <core mismatches +1>, -l <core length>, -e <total mismatches * 30 + 30> and—y. Subsequently, the output from Bowtie is parsed and only alignments including a proper PAM are listed.

### Off-target mismatch score

For each off-target site of any sgRNA a score is computed that indicates the likelihood of a stable sgRNA/DNA heteroduplex. Based on experimental evidence this likelihood decreases the closer the mismatch is to the PAM [13–15]. This finding is quantified according to the following formula

$$score_{off-target}=\sum_{mismatch}1.2^{pos},$$

where *pos* is the position of each mismatch, counted from the 5’ end. The base of the power expression was determined empirically.

### Assignment of closest gene to off-target sites

To handle the files containing the exon coordinates for each organism (bed files), the python library bx-python (https://bitbucket.org/james_taylor/bx-python/) is used and the BedInterval class is extended. Only exons closer than 100kb to the predicted off-target sites are assigned, otherwise “NA” is given as output. If target site and exon coordinates overlap, the distance is assigned to 0.

For each species the coordinates and the corresponding gene name and identifier of annotated exons are obtained from ENSEMBL (version 77) [19] using the BioMart interface. For medaka, additional genes were included based on RNA-seq data from different embryonic stages (unpublished data).

### sgRNA target site score

The list of sgRNA target sites is ranked according to the number of predicted off-target sites and their potential deleterious effects on the respective off-target gene. The ranking is based on a single score that combines the number of off-target sites, the distribution of their mismatches and the distance to the closest annotated exon. This score is defined by the following equation:

$$score=\sum_{off-targets}\left[\frac{\log10(dist)+score_{off\_target}}{total\_off\_targets}\right]-total\_off\_targets,$$

where *dist* is the distance of each off-target site to the corresponding closest exon. For this score only off-target sites with an associated exon are considered.

### Ethics Statement

All fish are maintained in closed stocks at Heidelberg University. Medaka (*Oryzias latipes*) husbandry and experiments were performed according to local animal welfare standards (Tierschutzgesetz §11, Abs. 1, Nr. 1, husbandry permit number 35–9185.64/BH Wittbrodt and mutagenesis permit number G-206/09) and in accordance with European Union animal welfare guidelines. The fish facility is under the supervision of the local representative of the animal welfare agency. Embryos of medaka of the wildtype Cab strain were used at stages prior to hatching. Medaka were raised and maintained as described previously [20]. Lines used in the study were medaka wildtype Cab and *Wimbledon* ^*-/+*^ [21].

### sgRNA target site selection

With CCTop, sgRNA target sites (pattern: N20NGG, core length = 12, max. core mismatches = 2, max. total mismatches = 4) were selected according to their best hit/least off-target. For efficient *in vitro* transcription from the T7 promoter, GG is necessary at the 5’ end of the respective sgRNAs. If not contained in the genomic target sequence, CCTop offers either extension or substitution of the most 5’ nucleotide(s) of the suggested primers [7,8] for sgRNA cloning (small g) to yield the 5’ leading GG necessary for *in vitro* transcription: sgRNA-1 target site (*eGFP*) 5’-GGCGAGGGCGATGCCACCTACGG-3’, sgRNA-2 target site (*cryaa*) 5’-GGTCAGGGTCAGCAGTCCATCGG-3’, sgRNA-3 target site (*rx2*) 5’-GCATTTGTCAATGGATACCCTGG-3’ and sgRNA-4 target site (*actb*) 5’-GGATGATGACATTGCCGCACTGG-3’.

### Cas9 mRNA and sgRNA generation

The plasmid JDS246 (Cas9; addgene: #43861) was linearized with *MssI*-FD (Thermo Scientific) and *in vitro* transcribed with T7 Ultra Kit (Ambion). After polyadenylation, mRNA was purified with RNeasy Mini Kit (Qiagen). sgRNA plasmids were generated via oligo annealing (sgRNA-1_F 5’-TAGGCGAGGGCGATGCCACCTA-3’, sgRNA-1_R 5’-AAACTAGGTGGCATCGCCCTCG-3’; sgRNA-2_F 5’-TAGGTCAGGGTCAGCAGTCCAT-3’, sgRNA-2_R 5’-AAACATGGACTGCTGACCCTGA-3’; sgRNA-3_F 5’-TAgGCATTTGTCAATGGATACCC-3’, sgRNA-3_R 5’-AAACGGGTATCCATTGACAAATG-3’; sgRNA-4_F 5’-TAGGATGATGACATTGCCGCAC-3’, sgRNA-4_R 5’-AAACGTGCGGCAATGTCATCAT-3’) and subsequently ligated into *BsaI* (Thermo Scientific) digested vector DR274 (addgene: #42250). The template for *in vitro* transcription was released from purified plasmid with *DraI*-FD (Thermo Scientific) and transcribed with T7 MAXIscript Kit (Ambion) or T7 MEGAshortscript Kit (Ambion). Purification was performed via ammonium acetate precipitation and phenol/chloroform extraction following the manufacturer’s guidelines. Cas9 mRNA and sgRNAs were stored at -80°C.

### *In vitro* cleavage assay with Cas9 protein

DNA cleavage assay was carried out based on [3] with commercially available Cas9 enzyme (NEB). PCR amplified genomic fragments for each sgRNA-1 off-target site (OT#1_F 5’-AGAGGCAAGTAAAGGTCAAGTAGG-3’, OT#1_R 5’-TCACATTGCAATGATGAGCACTTT-3’; OT#2_F 5’-CCAGCTCATGTTGAAAAGACACAT-3’, OT#2_R 5’-CCCCCACAGATGAAATGAAAAGAC-3’; OT#3_F 5’-TACCCAAAAATTGTAAGCCAGCAG-3’, OT#3_R 5’-AGATCTGATCCGGTTTCAAAGTGA-3’) were cloned into pGEM-T easy vector (Promega). The plasmids were pre-linearized with *BsaI* (NEB) about 2kb from the sgRNA target site. 3nM of the pre-linearized plasmids were incubated for one 1h with 30nM sgRNA-1 and 30nM Cas9 protein (NEB) and supplemented with Cas9 nuclease buffer (NEB) in a 30μl reaction volume at 37°C. Gel electrophoresis was performed on 1.5% agarose gel in 1x TAE buffer (40mM Tris, 20mM acetic acid, 1mM EDTA).

### Microinjections and screening of embryos

Embryos were injected at one-cell stage according to [22]. The following concentrations were used: Cas9 mRNA between 150 and 300ng/μl, sgRNAs 15ng/μl, and plasmid donors 8-10ng/μl. All components were diluted in nuclease-free ddH_2_O (Sigma). Dead specimens were removed and from two days post fertilization onwards embryos were screened for eGFP expression. To acquire images of eGFP expressing embryos, either a SMZ18 fluorescence-screening binocular (Nikon) or an AZ100 (Nikon) was used. Maximum Z-projections of stacks were generated in Fiji [23].

### Donor construction

For *in vivo* linearization of the donor plasmids, sgRNA-1 target site (T1) was cloned into the Golden GATEway cloning system [24] via oligo annealing (T1_F 5’-GATCAGGCCTGCAGCTGGGCGAGGGCGATGCCACCTACGGCTCGAGCTCGTAC-3’, T1_R 5’-GAGCTCGAGCCGTAGGTGGCATCGCCCTCGCCCAGCTGCAGGCCT-3’).

Homology flanks were selected according to integration sites and PCR amplified with primers extended with *BamHI* (forward primer) or *KpnI* (reverse primer) restriction sites via Q5 polymerase (NEB) from wildtype medaka genomic DNA (*actb* 5’ homology flank: F 5’-GGGGATCCCAGCAACGACTTCGCACAAA-3’, R 5’-GGGGTACCGGCAATGTCATCATCCATGGC-3’; *rx2* 5’ homology flank: F 5’-GCCGGATCCAAGCATGTCAAAACGTAGAAGCG-3’, R 5’-GCCGGTACCCATTTGGCTGTGGACTTGCC-3’). *eGFP* ^*var*^ was generated via fusion PCR (fragment 1 eGFP_F 5’-GCCGGATCCGGAGTGAGCAAGGGCGAGGAGCT-3’, eGFPvar_R 5’-GTACGTCGCGTCACCTTCACCCTCGCCGGAC-3’; fragment 2 eGFPvar_F 5’-TGAAGGTGACGCGACGTACGGCAAGCTGACCCTG-3’, eGFP_R 5’-GCCGGTACCTCCCTTGTACAGCTCGTCCATGCC-3’) with Q5 polymerase (NEB) on an *eGFP* template. *eGFP* forward and reverse primers were extended with *BamHI* or *KpnI* restriction sites, respectively for cloning via Golden GATEway.

## Results and Discussion

### CCTop—CRISPR/Cas9 target online predictor

**Fig 1:**
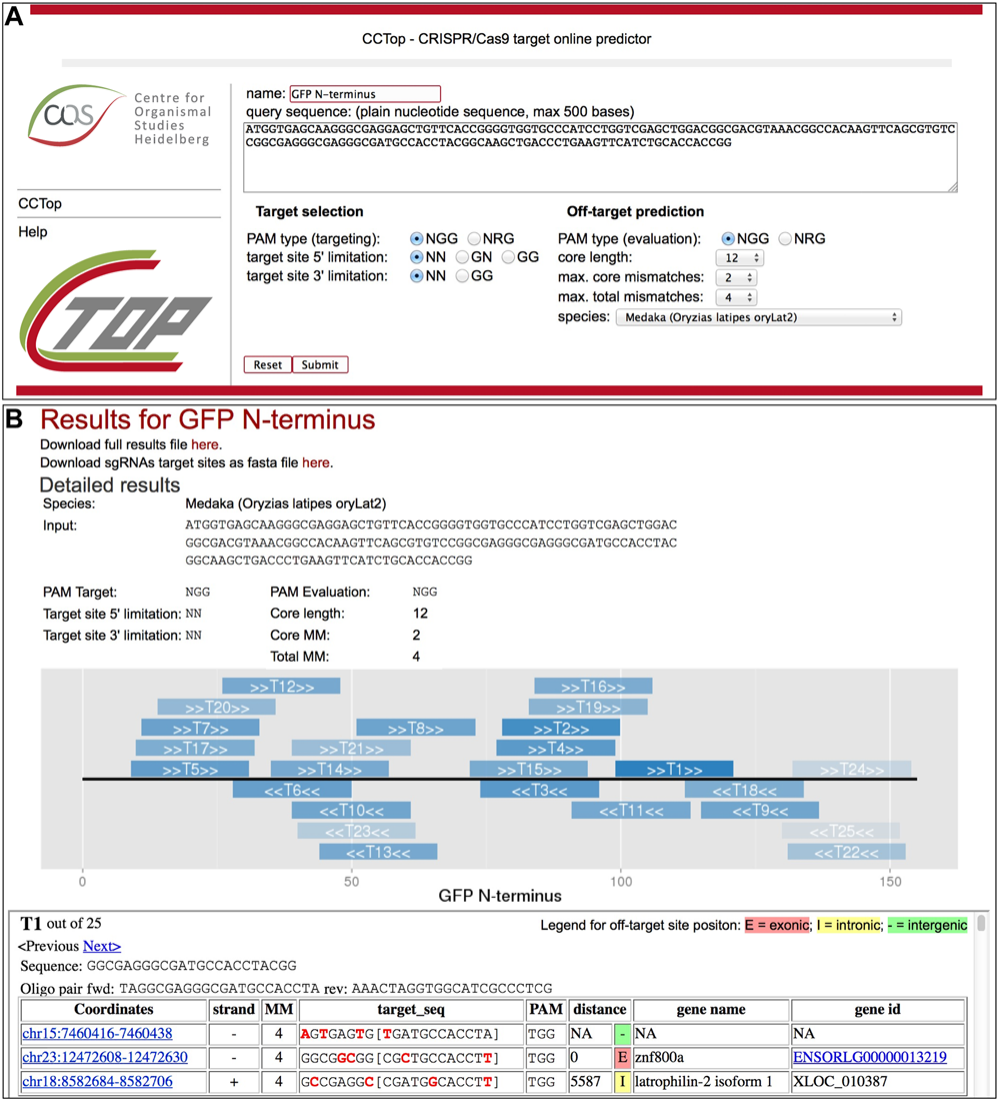
CCTop web interface. (A) Main page containing the input fields to customize the identification of sgRNA target sites and the off-target prediction. (B) Results page providing detailed information of all identified sgRNA target sites.

To overcome the aforementioned limitations of currently available CRISPR prediction tools, we designed the CRISPR/Cas9 target online predictor—CCTop—(crispr.cos.uni-heidelberg.de) as a web tool with a user friendly and intuitive interface (Fig 1A). From any provided DNA sequence all sgRNA target sites will be identified according to adjustable parameters like the type of PAM (‘NGG’ or ‘NRG’), the identity of the two most 5’ nucleotides (‘NN’, ‘GN’ or ‘GG’) [8,25] as well as the two most 3’ nucleotides (‘NN’ or ‘GG’) [26].

For off-target predictions, the selection of a PAM type can be made separately. Experimental evidence indicates that Cas9 nuclease activity strongly correlates with the mismatch position along the sgRNA/DNA heteroduplex. Mismatches close to the PAM will most likely abolish the introduction of a DSB, while more distal mismatches are tolerated [13–15].

We incorporate these findings as a simplified parameter, and define the nucleotides adjacent to the PAM as core sequence (12bp default length). More than two mismatches in that core abolish DSB introduction [13–15]. Furthermore, sites with more than four mismatches are not targeted by sgRNA/Cas9 [13,15,27]. As these options are based on current knowledge, future improvements in the field will be implemented and specified in the ‘help’ section of the webpage.

At the moment, CCTop provides sgRNA evaluation on the human (*Homo sapiens* GRCh38/hg38), mouse (*Mus musculus* GRCm38/mm10), medaka (*Oryzias latipes* oryLat2), *Xenopus tropicalis* (JGI4.2/xenTro3), zebrafish (*Danio rerio* Zv9/danRer7), stickleback (*Gasterosteus aculeatus* BROADS1/gasAcu1), cavefish (*Astyanax mexicanus* AstMex102), *Caenorhabditis elegans* (WBcel235), Drosophila (*D*. *melanogaster* BDGP5/dm3) and *Arabidopsis thaliana* (TAIR10) genomes. This list will regularly be updated and extended to more organisms in response to community requests.

After processing, a results page (Fig 1B) is displayed containing the input parameters, a graphical representation of the query sequence with the identified sgRNA target sites as well as a full list of all candidates ranked by taking into account the number of total off-target sites, the distribution of mismatches and the proximity to exons. For each sgRNA target site, cloning oligonucleotides are provided for the DR274 sgRNA vector [7]. Detailed information is provided for each potential off-target site: genomic coordinates, target sequence with highlighted mismatches, distance and position (exonic, intronic or intergenic) in respect to the closest exon and its corresponding name and identifier. If applicable, the off-target site coordinates are linked to the UCSC Genome Browser [28], while gene identifiers are linked to ENSEMBL [19]. If the query sequence belongs to the selected species, a link to the UCSC Genome Browser is provided for enhanced visualization of the query sequence and target site distribution in addition to other genomic or epigenetic features. Moreover, a fasta file containing all sgRNA target sites as well as a tab separated file containing the full results can be downloaded.

CCTop provides all the information necessary to swiftly identify the best candidate sgRNA represented by the order of the sgRNA target sites. Depending on the goal of the experiment, e.g. gene knock-out or knock-in, the best suited candidate might not always be the top hit. Hence, we encourage the user to explore the full list.

### Experimental validation of sgRNAs predicted by CCTop

**Fig 2:**
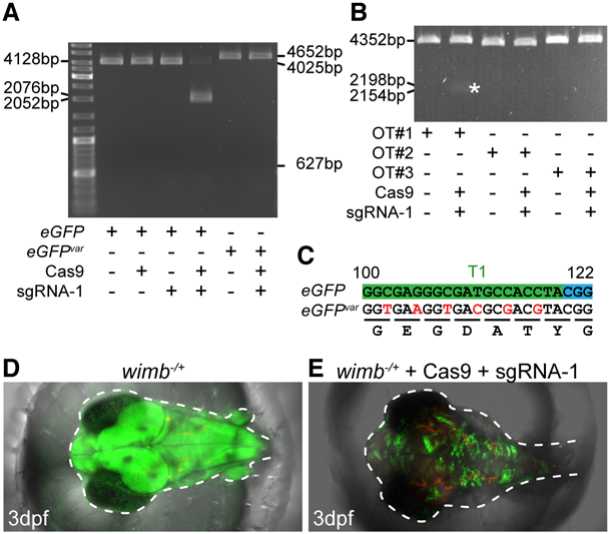
Experimental verification of sgRNA-1. (A) *In vitro* cleavage depending on sgRNA-1/Cas9 occurred on linearized plasmids containing *eGFP* but not *eGFP* ^*var*^. Successful cleavage of *eGFP* plasmid (4128bp) resulted in a 2052bp and 2076bp fragment. The absence of expected fragments (627bp, 4025bp) demonstrated that *eGFP* ^*var*^ (4652bp) was not digested by sgRNA-1/Cas9. (B) A faint double band (2154bp, 2198bp, asterisk) indicated inefficient digestion of off-target 1 (OT#1) while OT#2 and OT#3 (S1 Table) were not cleaved. Note: contrast was enhanced for better visualization. (C) Silent mutations of sgRNA-1 target site in *eGFP* ^*var*^. (D-E) Injections of sgRNA-1 and Cas9 mRNA into *wimb* ^*-/+*^ embryos (D) resulted in strong inactivation of eGFP (E).

We applied CCTop to determine the most specific sgRNA target site (T1) within the N-terminus of *eGFP* in the context of the medaka (*Oryzias latipes*) genome (PAM type ‘NGG’ for targeting and evaluation, core length = 12, max. core mismatches = 2, max. total mismatches = 4). To test the on-target efficacy of sgRNA-1 against T1, we performed an *in vitro* assay. Incubation of sgRNA-1, Cas9 protein and an *eGFP* containing plasmid revealed efficient cleavage of the template DNA (Fig 2A). To test the specificity of sgRNA-1, a variant of *eGFP* (*eGFP* ^*var*^) was used containing six silent mutations in T1 (Fig 2A, 2C). This modification prevented sgRNA-1 from targeting *eGFP* ^*var*^ (Fig 2A).

CCTop predicted three potential off-target sites for the sgRNA-1 in the medaka genome with different distributions of four mismatches relative to the corresponding PAMs (Fig 1B and S1 Table). We performed the *in vitro* digestion on all three loci (Fig 2B). Limited Cas9-dependent digestion was detected for off-target number 1 (OT#1, Fig 2B, asterisk), but neither for OT#2 nor for OT#3. The first mismatch in OT#1 appears at position twelve from the PAM (Fig 1B and S1 Table), experimentally corroborating the ranking criteria of CCTop.

To validate the efficacy of sgRNA-1 *in vivo*, microinjections into medaka zygotes of a ubiquitously expressing *eGFP* line were performed (*wimbledon*, *wimb*; Fig 2D) [21]. Cytoplasmatic injections of Cas9 mRNA and sgRNA-1 at the one cell stage resulted in efficient inactivation of *eGFP*. Besides sparse, residual *eGFP* expression no further phenotype was recognizable (Fig 2E). Randomly picked and sub-cloned amplicons of an injected embryo revealed the efficient mutation of *eGFP* in the *wimb* line (12/12) by the introduction of insertions/deletions (indels) or nucleotide substitutions at the T1 site (Fig 2E and S2 Fig).

**Fig 3:**
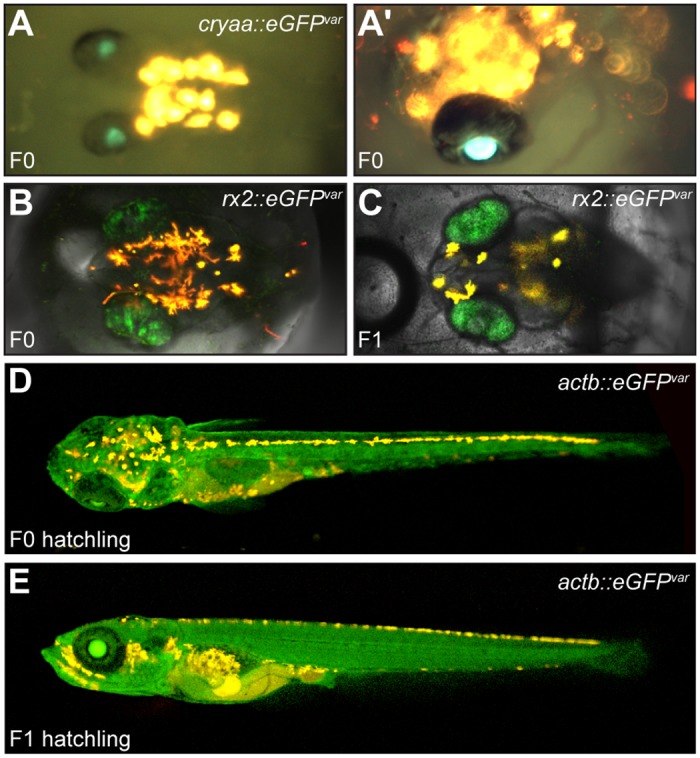
Visual evaluation of targeting specificity of selected sgRNAs. Exclusive and homogenous expression of eGFP in the domains of *cryaa* (A, A’), *rx2* (B) and *actb* (D) was evident already in the injected generation (F0). The integrations were transmitted to the next generation (F1; C, E).

In order to further validate the selection of sgRNAs by CCTop, we chose to target three endogenous genes with distinct expression patterns in medaka. *Alpha a crystallin* (*cryaa*) expression is restricted to the lens [29], the *retinal homeobox gene 2* (*rx2*) is exclusively expressed in the neuroretina of developing medaka [30] and *β-actin* (*actb*) is expressed ubiquitiously in the whole body [31]. For each gene we used CCTop to predict reliable sgRNAs targeting the immediate downstream sequence of the corresponding translational start site (S1 Table). For efficient screening of successful sgRNA/Cas9 targeting already in the injected generation, we generated a donor construct containing the T1 site for *in vivo* linearization and the *eGFP* ^*var*^ sequence. Upon co-injection of the donor plasmid, Cas9 mRNA, sgRNA-1 and the sgRNA against *cryaa*, *eGFP* ^*var*^ was integrated into the target locus via NHEJ (Fig 3A) [32,33]. In the cases of *rx2* and *actb*, homology flanks (ca. 400bp) were added for the *eGFP* ^*var*^ cassette for integration via HDR (Fig 3B, 3D) [5,34–36].

For each of the three candidate genes, eGFP has been consistently detected exclusively in the expected tissue in all experiments (Fig 3 and S2 Table). Interestingly, already the injected generation revealed highly homogeneous expression of *eGFP* ^*var*^ in the respective tissues/organs (Fig 3). After injection, visual detection of eGFP in the *cryaa* domain was low, due to the likely out-of-frame integration via NHEJ (4/422) (S2 Table). In contrast, the HDR-mediated integrations into the *rx2* and *actb* loci reached significantly higher rates, 72/472 and 79/270 respectively (S2 Table). Furthermore, the *eGFP* ^*var*^ integration into the *rx2* and *actb* loci was transmitted successfully to the next generation (*cryaa* was not pursued further). From 6 *rx2*::*eGFP* ^*var*^ positive fish, 5 transmitted the integration to the next generation (22.5% maximal germline transmission rate) (Fig 3C). For the *actb*::*eGFP* ^*var*^ positive fish, 2 out of 7 were founders (15.3% maximal germline transmission rate) (Fig 3E). The high integration rates of *eGFP* ^*var*^ and the strong specificity of the expression pattern validate that the sgRNAs identified by CCTop efficiently targeted their designated loci and that no off-target site interaction occurred.

The application of the CRISPR/Cas9 system is enhanced by a careful and precise off-target prediction. We provide an online tool matching the needs of both beginners and experts. This is achieved by a concise but complete number of selectable parameters. We provide validated default settings with high success rates in multiple experiments that can still be tuned. The top ranked sgRNA target sites have been experimentally validated *in vitro* and *in vivo* in different approaches. Taken together CCTop allows target selection in a wide range of model and non-model genomes and guides the user towards selecting the optimal target site.

## Supporting Information

S1 Fig Workflow of CCTop. The input sequence is scanned to identify sgRNA target sites according to the parameters specified in the main page. Oligo pairs for target site cloning are generated (see Material and Methods). For each candidate target site, the potential off-target sites are determined using Bowtie1. The closest exon is assigned to each potential off-target and its score is computed. With this information each candidate is ranked and finally the results are provided in different output formats. If the query sequence was derived from the same genome the candidate target sites were evaluated against, a bed-file containing the genomic coordinates and target scores is passed on to the UCSC genome browser as custom track.
(TIF)

S2 Fig Mutations in *eGFP* induced by sgRNA-1/Cas9. Sequencing of the target site of sgRNA-1/Cas9 mRNA injected *wimb* ^*-/+*^ specimen (Fig 2E) revealed indel formation/nucleotide substitution in all sub-cloned *eGFP* sequences. **Δ**, deletions (red dashes); i, insertions; s, substitutions (purple). Black background indicates premature STOP codon.
(TIF)

S1 Table Detailed information on the selected sgRNA targets from CCTop results files. (XLSX)

S2 Table Screening results of injection experiments. Targeted insertions of *eGFP* ^*var*^ into the *cryaa*, *rx2* and *actb* loci.
(DOCX)
