# Correction: CCTop: An Intuitive, Flexible and Reliable CRISPR/Cas9 Target Prediction Tool

**DOI:** 10.1371/journal.pone.0176619

**Published:** 2017-04-20

**Authors:** Manuel Stemmer, Thomas Thumberger, Maria del Sol Keyer, Joachim Wittbrodt, Juan L. Mateo

The first sentence in the Material and Methods section under the subheading "Off-target search" is incorrect. The correct sentence is: For each off-target site of any sgRNA a score is computed that indicates the likelihood of an unstable sgRNA/DNA heteroduplex.
