# Supplementary figures and images for: CCTop: An Intuitive, Flexible and Reliable CRISPR/Cas9 Target Prediction Tool
Source: PLoS One. 2015 Apr 24;10(4):e0124633. doi: 10.1371/journal.pone.0124633 (PMC4409221; doi:10.1371/journal.pone.0124633)

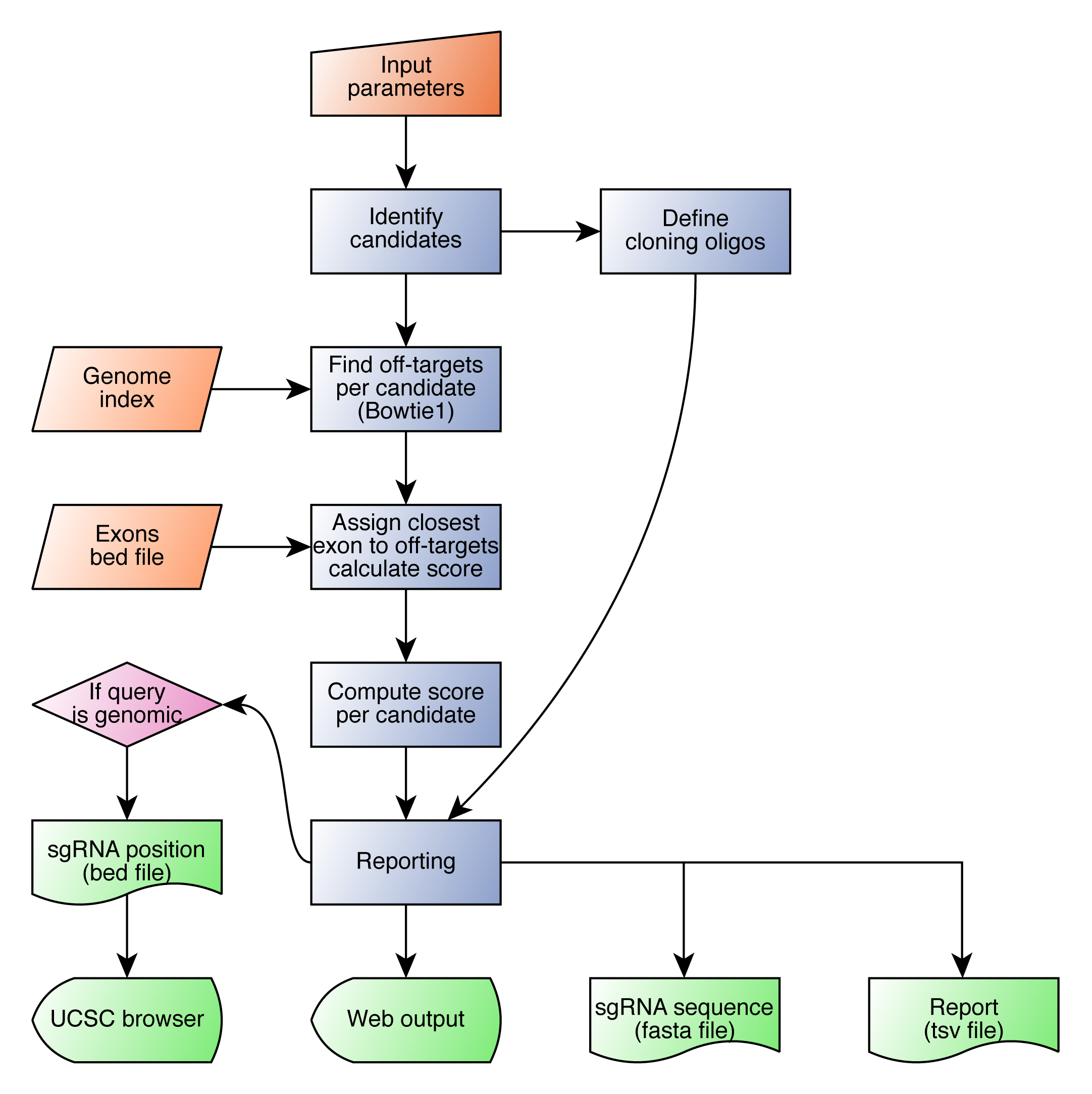

Supplement: S1 Fig — The input sequence is scanned to identify sgRNA target sites according to the parameters specified in the main page. Oligo pairs for target site cloning are generated (see Material and Methods). For each candidate target site, the potential off-target sites are determined using Bowtie1. The closest exon is assigned to each potential off-target and its score is computed. With this information each candidate is ranked and finally the results are provided in different output formats. If the query sequence was derived from the same genome the candidate target sites were evaluated against, a bed-file containing the genomic coordinates and target scores is passed on to the UCSC genome browser as custom track. (TIF) [file pone.0124633.s001.tif]

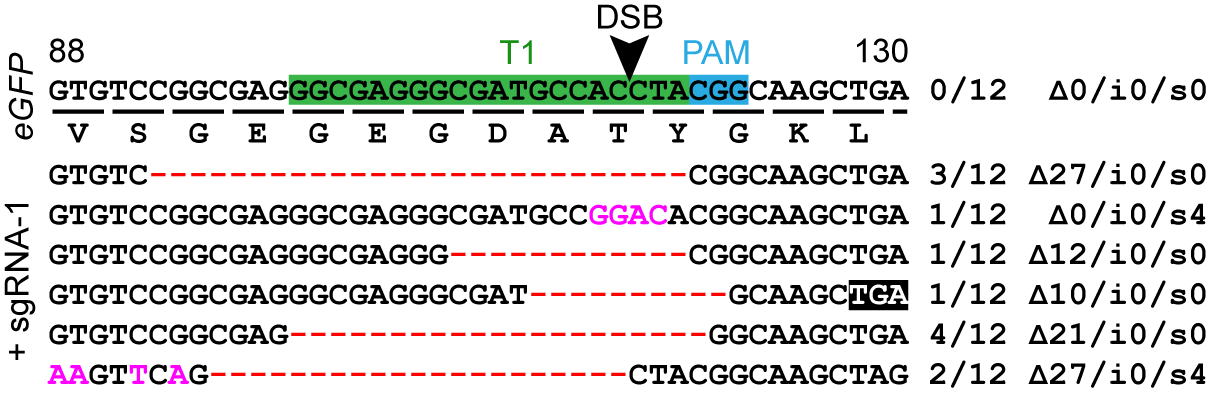

Supplement: S2 Fig — Sequencing of the target site of sgRNA-1/Cas9 mRNA injected wimb -/+ specimen (Fig 2E) revealed indel formation/nucleotide substitution in all sub-cloned eGFP sequences. Δ, deletions (red dashes); i, insertions; s, substitutions (purple). Black background indicates premature STOP codon. (TIF) [file pone.0124633.s002.tif]
