# Supplementary material for: CCTop: An Intuitive, Flexible and Reliable CRISPR/Cas9 Target Prediction Tool
Source: PLoS One. 2015 Apr 24;10(4):e0124633. doi: 10.1371/journal.pone.0124633 (PMC4409221; doi:10.1371/journal.pone.0124633)
Supplement: S2 Table — Targeted insertions of eGFP var into the cryaa, rx2 and actb loci. (DOCX) [file pone.0124633.s004.docx]

**Table S2.**

|  | ***cryaa*** | | | | ***rx2*** | | | | | ***actb*** | | |
| --- | --- | --- | --- | --- | --- | --- | --- | --- | --- | --- | --- | --- |
| **dead** | 8 | 27 | 11 | 30 | 29 | 62 | 56 | 38 | 68 | 34 | 0 | 32 |
| **GFP** | 1 | 0 | 2 | 1 | 14 | 19 | 13 | 14 | 12 | 25 | 19 | 35 |
| **no GFP** | 54 | 148 | 57 | 159 | 111 | 47 | 68 | 80 | 78 | 39 | 60 | 87 |
| **deformed** | 0 | 0 | 0 | 0 | 1 | 0 | 3 | 7 | 5 | 0 | 0 | 5 |
| **sum** | 63 | 175 | 70 | 190 | 155 | 128 | 140 | 139 | 163 | 98 | 79 | 159 |
